# Stability of an Electrodeposited Nanocrystalline Ni-Based Alloy Coating in Oil and Gas Wells with the Coexistence of H_2_S and CO_2_

**DOI:** 10.3390/ma10060632

**Published:** 2017-06-09

**Authors:** Yiyong Sui, Chong Sun, Jianbo Sun, Baolin Pu, Wei Ren, Weimin Zhao

**Affiliations:** 1 School of Petroleum Engineering, China University of Petroleum, Qingdao 266580, China; suiyy@upc.edu.cn; 2 School of Mechanical and Electronic Engineering, China University of Petroleum, Qingdao 266580, China; sunchongupc@126.com (C.S.); zhaowm@upc.edu.cn (W.Z.); 3 Shengli Oilfield Shengxin Antisepsis Co., Ltd., Dongying 257091, China; dysxpu123@163.com (B.P.); renweizhenzhen@163.com (W.R.)

**Keywords:** Ni-based alloy, nanocrystalline coating, electrodeposition, H_2_S/CO_2_ corrosion, corrosion scale

## Abstract

The stability of an electrodeposited nanocrystalline Ni-based alloy coating in a H_2_S/CO_2_ environment was investigated by electrochemical measurements, weight loss method, and surface characterization. The results showed that both the cathodic and anodic processes of the Ni-based alloy coating were simultaneously suppressed, displaying a dramatic decrease of the corrosion current density. The corrosion of the Ni-based alloy coating was controlled by H_2_S corrosion and showed general corrosion morphology under the test temperatures. The corrosion products, mainly consisting of Ni_3_S_2_, NiS, or Ni_3_S_4_, had excellent stability in acid solution. The corrosion rate decreased with the rise of temperature, while the adhesive force of the corrosion scale increased. With the rise of temperature, the deposited morphology and composition of corrosion products changed, the NiS content in the corrosion scale increased, and the stability and adhesive strength of the corrosion scale improved. The corrosion scale of the Ni-based alloy coating was stable, compact, had strong adhesion, and caused low weight loss, so the corrosion rates calculated by the weight loss method cannot reveal the actual oxidation rate of the coating. As the corrosion time was prolonged, the Ni-based coating was thinned while the corrosion scale thickened. The corrosion scale was closely combined with the coating, but cannot fully prevent the corrosive reactants from reaching the substrate.

## 1. Introduction

According to the statistics, oil well tubes account for about 40% of the total steel consumption in the oil and gas industry. More than half of oil well tubing failures are caused by corrosion-related problems [1]. Currently, about 1/3 of oil and gas fields in the world contain H_2_S. For example, the newly developed oil and gas fields in Sichuan and Xinjiang in China have very high contents of H_2_S and CO_2_, and in certain regions, such contents even exceed 10%. Therefore, the H_2_S/CO_2_ corrosion of oil tubes has become an increasingly prominent problem [2,3,4,5,6]. The most common anti-corrosion measure is to adopt anti-corrosive metals for oil and gas tubes. Stainless steel tubing is generally preferred for deep gas wells in an environment without H_2_S. However, for the sour gas field that contains H_2_S, the resistance to sulfide stress corrosion cracking (SSCC) shall also be taken into consideration in addition to the corrosion weight loss. According to the practice recommended by the ISO 15156 standard [7], the highest partial pressure of H_2_S applicable to super 13Cr stainless steel is 0.01 MPa, and that to duplex stainless steel is 0.1 MPa [7]. Therefore, Ni-based alloys are the only option for the anti-corrosive oil tubes used in the sour oil and gas fields with a relatively higher partial pressure of H_2_S, and this will inevitably bring about higher material costs. If the anti-corrosive process of “carbon steel or low-alloy steel + corrosion inhibitor” is adopted, the production and management costs will be greatly increased [8,9].

In some corrosive environments, the economic cost and operation cost can be well balanced by coating carbon steels or low-alloy steels to improve their corrosion resistance [10,11]. The Ni-based alloy coatings (such as Ni-W and Ni-W-P coatings, etc.) are characterized by good corrosion resistance [11,12,13,14,15,16,17,18,19,20,21,22,23], wear resistance [13,16,20,21,23,24], high hardness [13,17,21,22,25], environmental friendliness [14], etc., and can replace traditional chrome coatings as well as thermal spraying coatings in surface protection [12,14,16,18,26,27,28]. In particular, the thermal spraying coatings inevitably have certain porosities [26,27,28], which pose great corrosion risks to the substrate in oil and gas environments. Electrochemical measurement technology is the most common method employed in the study of corrosion resistance of Ni-based alloy coatings [11,12,15,18,19,20,22]. For example, the instantaneous corrosion rate and passivation property of the coating can be obtained rapidly by measuring the polarization curve [11,12,15,18,19,20,22]. However, the electrochemical test cannot accurately reflect the long-term service performance of the coating in an actual environment. Currently, the Ni-based alloy coating tubings have been used in several oil fields in China, and they exhibit excellent CO_2_ corrosion resistance [29,30], as well as good scaling resistance and wear resistance [30]. However, there is still very limited research on the corrosion of Ni-based alloy coating tubings in the environment with the coexistence of H_2_S and CO_2_, especially in the sour oil and gas fields with a high partial pressure of H_2_S, and this restricts the further promotion and application of Ni-based alloy coating tubings. The aim of this work is to investigate the stability of the Ni-based alloy coating and the characteristics of the corrosion scale in a H_2_S/CO_2_ environment with high temperature and high pressure, providing technical support for its application in a H_2_S/CO_2_ environment.

## 2. Experimental Methods

### 2.1. Materials and Coating Preparation

The Ni-based alloy coating was prepared on a N80 steel substrate by means of a DC electrodeposition method according to the patent technology of Shengli Oilfield Shengxin Antisepsis Co., Ltd. (Dongying, China) [31], which mainly included five processes: degreasing, pickling, activation, plating, and heat treatment (200–400 °C, 2–5 h). An iridium coating titanium electrode was used as the anode. N80 steel, with a composition (mass fraction) of 0.29% C, 0.25% Si, 1.38% Mn, 0.016% P, 0.002% S, 0.037% Cr, 0.002% Ni, 0.009% Cu, and Fe balance, was machined into a size of 50 mm × 10 mm × 3 mm, which was used as the cathode. Prior to the electrodeposition process, the N80 steel surface was abraded with SiC paper of decreasing roughness (up to 2000 grit). Table 1 gives the composition of the sulfate-citrate acid plating bath and the operational parameters used to electrodeposit the Ni-W-P coating.

BG90SS anti-sulfide tubing (Baoshan Iron & Steel Co., Ltd., Shanghai, China) was used to compare and evaluate the corrosion resistance of the Ni-based alloy coating. BG90SS anti-sulfide tubing, with a composition (mass fraction) of 0.29% C, 0.25% Si, 0.60% Mn, 0.009% P, 0.002% S, 1.04% Cr, 0.037% Ni, 0.32% Mo, 0.042% Cu, 0.034% Al, 0.027% Ti, 0.003% V, and Fe balance, was machined into a size of 50 mm × 10 mm × 3 mm. The working surface of each specimen was abraded with SiC paper of decreasing roughness (up to 1000 grit), rinsed with deionized water, and degreased with acetone.

Prior to the tests, the four parallel specimens for each weight loss test were weighed using an electronic balance with a precision of 0.1 mg, and were then stored in a desiccator.

### 2.2. Potentiodynamic Polarization

An Interface 1000 electrochemical workstation (Gamry Instruments, Warminster, PA, USA) was used for electrochemical measurements. A three-electrode electrochemical cell was used with a platinum plate as a counter electrode and a saturated calomel electrode (SCE) as a reference electrode. BG90SS steel and the Ni-based alloy coating specimens were respectively employed as the working electrode (WE). After the WE was immersed in the solution for 30 min to obtain a stable open circuit potential (OCP), the potentiodynamic polarization curve was carried out in a range of −500 mV–1000 mV with respect to the corrosion potential, and with a scan rate of 0.5 mV/s. All the potentials in this study referred to this reference electrode.

The test solution to simulate the formation water from a gas condensate field in China was made up of analytical grade reagents and deionized water. The chemical composition of the test solution is listed in Table 2. Prior to the tests, the solution was purged with N_2_ (99.99%) for at least 4 h, and then the test solution was saturated with H_2_S/CO_2_ mixed gases at a speed of 200 mL/min for 1 h. Afterwards, the WE was immersed in the solution, and the H_2_S/CO_2_ mixed gases were then bubbled through the solution at a low flow rate of 20 mL/min. The tests were performed under static conditions at 25 °C and atmospheric pressure (H_2_S/CO_2_ pressure was 0.1 MPa).

### 2.3. Weight Loss Test

The weight loss tests were carried out in a 3 L autoclave to investigate the effect of temperature and corrosion time on the corrosion behavior of the Ni-based alloy coating in the H_2_S/CO_2_ environment, and the results were compared with those of BG90SS steel. The test conditions are listed in Table 3. Before the tests, the solution (Table 2) was purged with high purified N_2_ to deoxidize for 12 h. The specimens were immersed into the solution as soon as the solution was added into the autoclave, and then N_2_ purging was introduced to remove the air for 2 h immediately after the autoclave was closed. After that, the vent valve was closed. The solution was heated to the test temperature, and then H_2_S and CO_2_ gases were respectively injected into the autoclave to the desired pressure.

After the corrosion tests, the specimens were taken out of the autoclave, rinsed in deionized water, dehydrated in alcohol, and dried in air respectively. One of the four parallel specimens of each test was retained for surface characterization of the corrosion scale. The other three specimens were descaled in the solution consisting of hydrochloric acid (150 mL, density 1.19 g/mL) and deionized water (850 mL) at room temperature, and were then processed as above. After that, the specimens were weighed again to determine the weight loss. The corrosion rate was calculated through the following equation:

$$V_{CR}=\frac{8.76\times 10^{4}\Delta W}{S\rho t}$$

where *V_CR_* is the corrosion rate, mm/y; ∆*W* is the weight loss, g; *S* is the exposed surface area of the specimen, cm^2^; *ρ* is the density of the specimen, g/cm^3^; *t* is the corrosion time, h; and 8.76 × 10^4^ is the unit conversion constant. The average corrosion rate with error bars was calculated from the three parallel specimens for each test.

### 2.4. Adhesive Force Test of the Corrosion Scale

Excellent adhesive performance can help to enhance the protection of the corrosion scale to the metal substrate [8]. To test the adhesive strength of the corrosion scale on the coating surface, the tensile holder was machined into a rod of diameter 10 mm. The end face (mating surface) of the tensile rod was abraded with SiC paper (up to 1000 grit) until the surface roughness reached about 10 μm, and was then rinsed in deionized water, dehydrated in alcohol, and dried in air respectively. A TYBOND2178 adhesive (Shenzhen Tegu New Material Co., Ltd., Shenzhen, China) with tensile strength 20–30 MPa was used to connect the corrosion scale with the tensile holder. Prior to the tensile test, the adhesive was solidified for 24 h under ambient pressure and temperature. A WDML-5 tensile testing instrument (Beijing Heng Odd Instrument Co., Ltd., Beijing, China) was used for the tensile test. The loading rate was 1 mm/min. The tensile strength was measured when the corrosion scale was completely detached from the coating, which was considered as the adhesive force of the corrosion scale.

### 2.5. Morphological and Structural Characterization of the Ni-Based Alloy Coating and Corrosion Scale

The surface and cross-sectional morphologies of the Ni-based alloy coating and corrosion scale were observed using a scanning electron microscope (SEM) (JXA-8230, JEOL Ltd., Tokyo, Japan). The elemental compositions of the corrosion scale were analyzed using energy dispersive spectroscopy (EDS) (Inca X-act, Oxford instruments, Oxfordshire, UK) with an acceleration voltage of 15 kV. The phase compositions of the Ni-based alloy coating and corrosion scale were identified by means of X-ray diffraction (XRD) (Xpert Pro MPD, PANalytical B.V., Almelo, The Netherlands) with a Cu Kα X-ray source operated at 40 kV and 150 mA.

## 3. Results and Discussion

### 3.1. Morphology and Structure of the Electrodeposited Ni-Based Alloy Coating

**Figure 1:**
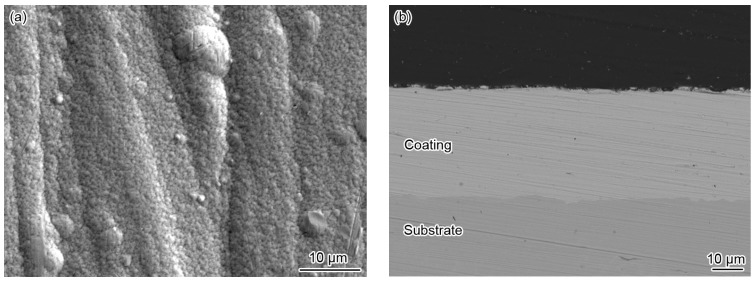
(**a**) SEM surface morphology and (**b**) cross-sectional backscattered electron image of the electrodeposited Ni-based alloy coating.

The surface of the prepared coating was homogeneous, bright, and free from cracks. The SEM surface and cross-sectional morphologies of the Ni-based alloy coating are shown in Figure 1. It can be seen that the coating presented a homogeneous fine granular morphology with a few globular nodules, and no cracks appeared (Figure 1a). The formation of a few globular nodules, which was a common morphology of the Ni-based alloy coating, was probably related to the composition of the plating solution [19,20,32,33]. Related literature suggested that these nodules diminished with the increase of the W content in the coating, and correspondingly increased its corrosion resistance [20]. In addition, the cross-sectional morphology showed that the coating with a thickness of about 37 μm was homogeneous, compact, and closely combined with the substrate (Figure 1b).

**Figure 2:**
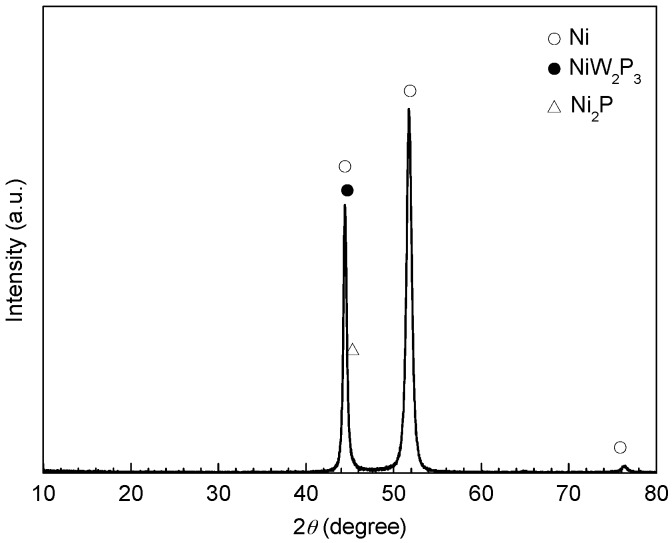
XRD spectra of the electrodeposited Ni-based alloy coating.

The phase structures of the electrodeposited Ni-based alloy coating after heat treatment were identified by means of XRD, as shown in Figure 2. It can be seen that the diffraction spectra of the Ni-based alloy coating showed sharp peaks at around 44.4° and 51.7°, characteristic of the (111) and (200) states of Ni according to standard reference card (No. 87-0712). It can be calculated that the grain sizes of Ni were respectively about 20.3 nm and 12.1 nm at 44.4° and 51.7°. However, NiW_2_P_3_ and Ni_2_P were also detected, but their diffraction peaks were not apparent when compared with that of Ni. This suggested that the nanocrystalline Ni-based alloy coating consisted of Ni crystals with small amounts of NiW_2_P_3_ and Ni_2_P.

### 3.2. Potentiodynamic Polarization Curve

**Figure 3:**
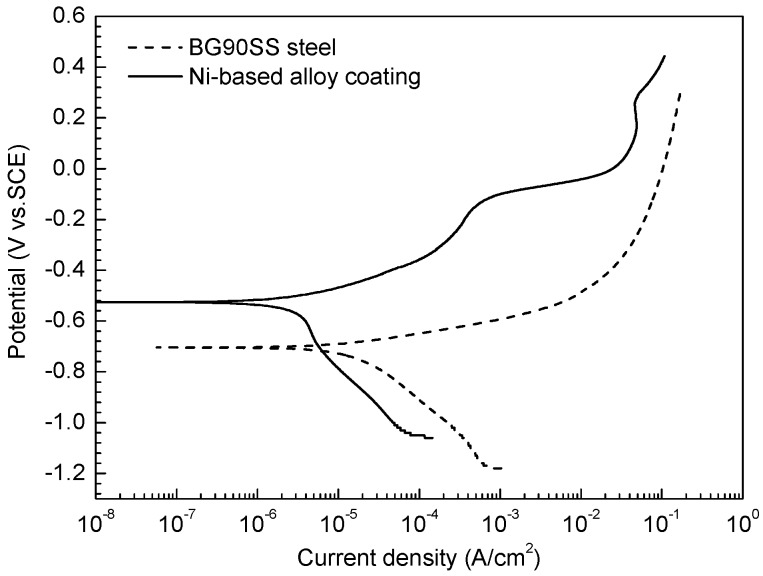
Polarization curves of the test materials in the simulated formation water at 25 °C and 0.1 MPa H_2_S/CO_2_.

Figure 3 shows the potentiodynamic polarization curves of the nanocrystalline Ni-based alloy coating and BG90SS steel exposed to the simulated formation water at 25 °C and 0.1 MPa H_2_S/CO_2_. Within the measurement range, the polarization curve of the nanocrystalline Ni-based alloy coating did not show obvious passivation, possibly because the hydrogen sulfide inhibited the formation of the oxide film on the coating surface [34]. In general, the higher the temperature of the medium is, the lower the pH of the medium is. Correspondingly, the metal passivation becomes more difficult. Therefore, it can be inferred that it was very difficult to form a passive film on the Ni-based alloy coating during the immersion tests with high temperature and high pressure (Table 3). Tafel’s extrapolation method was employed for determining the corrosion current density. The fitted values of the electrochemical parameters such as corrosion potential (*E_corr_*), corrosion current density (*i_corr_*), and the anodic and cathodic Tafel slopes (*b_a_* and *b_c_*) are listed in Table 3. Seen from Figure 3 and Table 4, the polarization curve of the Ni-based alloy coating exhibited an obvious positive shift compared with that of BG90SS steel, and correspondingly the *E_corr_* rose from −704 to −525 mV vs. SCE. Meanwhile, the cathodic and anodic polarization curves shifted to the left, and the corresponding Tafel slope was much larger than that of BG90SS steel. It is indicated that the anodic and cathodic processes of the Ni-based alloy coating were simultaneously suppressed under the test condition, and *i_corr_* dropped dramatically to a level which was 17% of that of the BG90SS steel. The above results suggested that the Ni-based alloy coating had a better H_2_S/CO_2_ corrosion resistance than the BG90SS steel in the H_2_S/CO_2_ environment.

### 3.3. Corrosion Rate and Morphology

**Figure 4:**
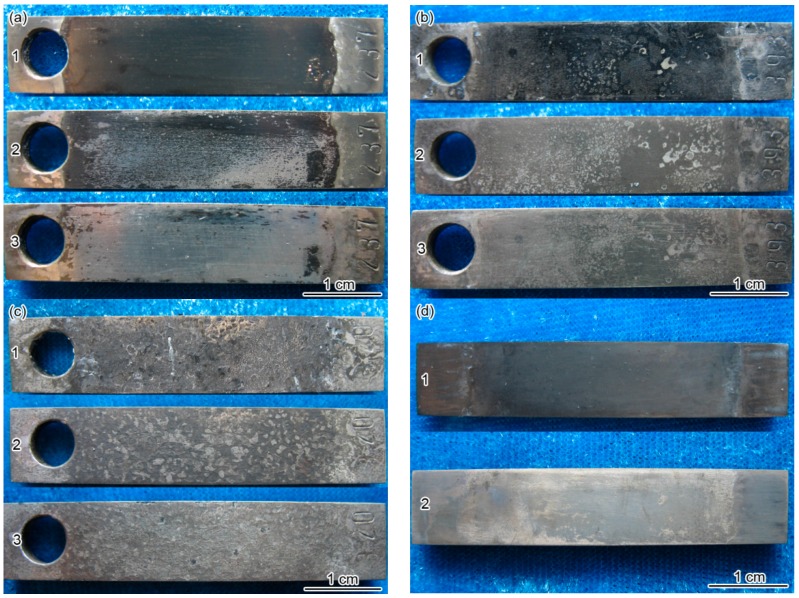
Macroscopic morphologies of (**a**–**c**) Ni-based alloy coating and (**d**) BG90SS steel exposed to the H_2_S/CO_2_ environment for 168 h at different temperatures: (**a**) 60 °C; (**b**,**d**) 90 °C and (**c**) 140 °C. (1—before pickling, 2—after pickling, and 3—after descaling by the mechanical method).

Figure 4 shows the macroscopic morphologies of the Ni-based alloy coating and BG90SS steel before and after removing the corrosion scales exposed to the H_2_S/CO_2_ environment for 168 h. It can be seen that the grey black corrosion scales with different color shades covered the specimen surface at different temperatures (Figure 4a1,b1,c1,d1), the corrosion scale on the Ni-based alloy coating was dense while that on the BG90SS steel was loose. After pickling, some mottled and broken corrosion products were still attached on the Ni-based alloy coating surface, which were hard to remove by acid pickling (Figure 4a2,b2,c2). This indicated that the corrosion scale formed on the Ni-based alloy coating had good acid resistance. By means of thermodynamic calculation, Ueda [35] found that in the 0.001 MPa H_2_S, 3.0 MPa CO_2_, and 0.1 MPa H_2_ saturated solution, the nickel sulfides were the most stable among the corrosion products, such as oxide, FeCO_3_, and other sulfides. These residual corrosion products that remained on the Ni-based alloy coating after pickling were likely related to the formation of stable Ni and S compounds. After further removing the residual corrosion products on the surface of the Ni-based alloy coating by using a hardwood stick, general corrosion morphologies were observed at different temperatures (Figure 4a3,b3,c3). However, the corrosion scale was very easily removed from the BG90SS steel surface by the acid pickling method. As exhibited in Figure 4d2, the BG90SS steel presented a general corrosion morphology.

**Figure 5:**
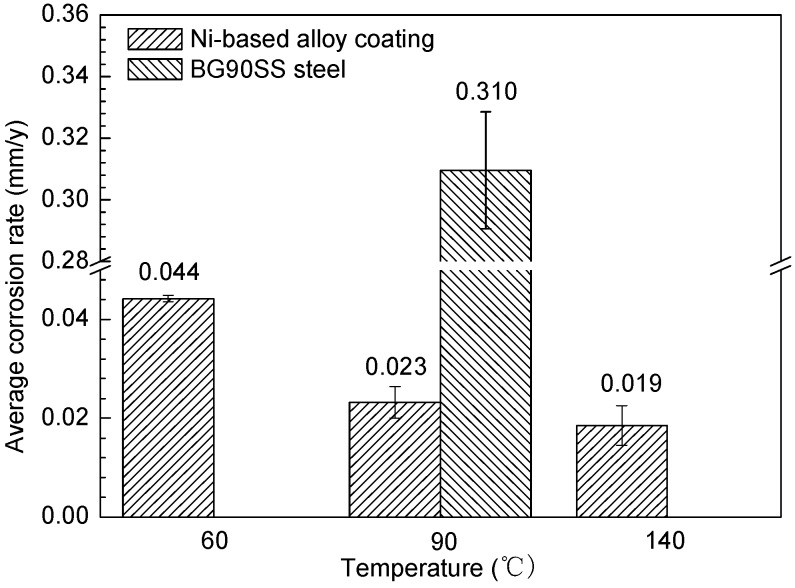
Corrosion rates of the Ni-based alloy coating and BG90SS steel exposed to the H_2_S/CO_2_ environment for 168 h at different temperatures.

Figure 5 shows the corrosion rates of the Ni-based alloy coating and BG90SS steel exposed to the H_2_S/CO_2_ environment for 168 h at different temperatures by using weight loss method. It can be seen that the corrosion rate of the coating decreased with an increase in the temperature, which was probably related to the protective improvement of the corrosion scale at high temperature conditions. Under the same temperature of 90 °C, the corrosion rate of the Ni-based alloy coating (0.023 mm/y) was far less than that of the BG90SS steel (0.310 mm/y); the former was only 7.4% of the latter. This suggested that the corrosion resistance of the Ni-based alloy coating was much higher than that of BG90SS steel, which was consistent with the electrochemical measurement results at room temperature (25 °C) and atmospheric pressure (0.1 MPa).

### 3.4. Effect of Temperature on the Characteristics of the Corrosion Scale

**Figure 6:**
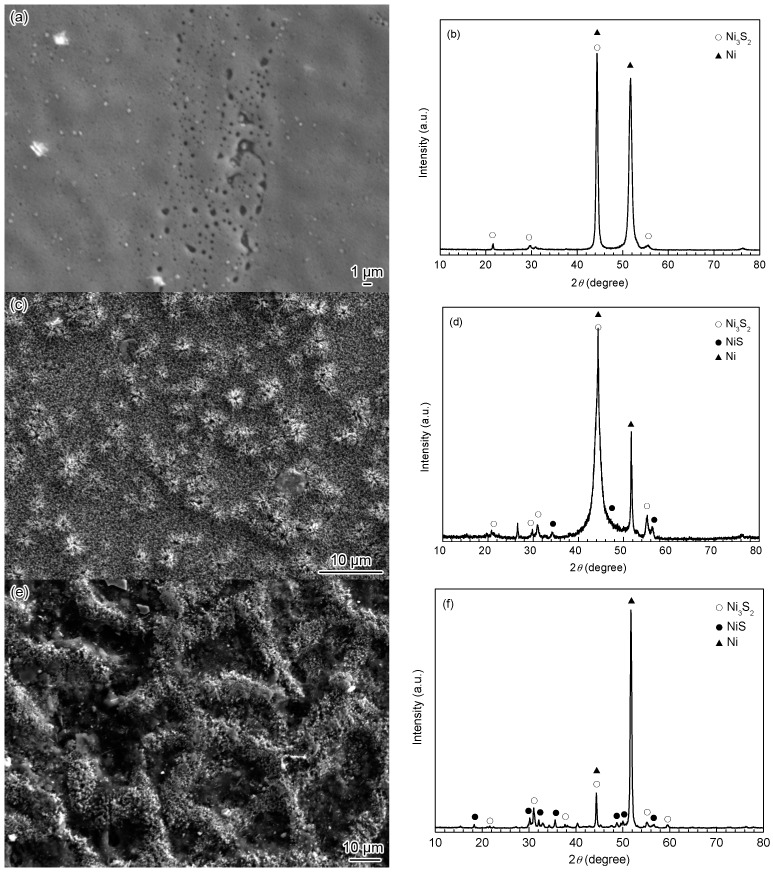
(**a**,**c**,**e**) SEM surface morphologies and (**b**,**d**,**f**) XRD spectra of corrosion scales on the Ni-based alloy coating exposed to the H_2_S/CO_2_ environment for 168 h at different temperatures: (**a**,**b**) 60 °C; (**c**,**d**) 90 °C and (**e**,**f**) 140 °C.

Figure 6 shows the SEM surface morphologies and XRD spectra of the corrosion scales on the Ni-based alloy coating after being corroded for 168 h at different temperatures. In view of the high sulfide formation capability of Ni [34], all the corrosion products were nickel sulfides within the range of experimental temperatures (Figure 6b,d,f), and thus the corrosion of the Ni-based alloy coating was mainly controlled by H_2_S corrosion. It should be noted that the diffraction peaks of Ni from the coating substrate were also detected in the XRD spectra, which were related to the thin corrosion scale formed on the coating surface. At 60 °C, the gel-like corrosion products with many holes distributed at local sites were observed, as shown in Figure 6a. The results of the XRD analysis revealed that the corrosion scale was mainly composed of Ni_3_S_2_ (Figure 6b). At 90 °C, tiny crystals stacked to form the corrosion scale, and many small cauliflower-like bulges were observed on the surface (Figure 6c). The XRD analysis revealed that the main components of the corrosion scale were Ni_3_S_2_ and small amounts of NiS (Figure 6d). At 140 °C, the corrosion scale formed on the Ni-based alloy coating which showed a coarse reticulate structure, with raised reticles, and sunken and rough meshes, and the coarse reticles were covered by tiny crystals (Figure 6e). The XRD analysis suggested that the corrosion scale was also mainly composed of Ni_3_S_2_ and NiS (Figure 6f). In addition, the diffraction peaks of NiS were obviously enhanced compared with that at 90 °C, indicating an increase in the NiS content. Zhao et al. [9] reported that the corrosion products of Ni-based alloy at 205 °C (1.5 MPa H_2_S and 3.5 MPa CO_2_) mainly consisted of NiS. It can be seen that the NiS content in the corrosion scale increased with the rising of the temperature, correspondingly, the composition and structure of the corrosion scale also changed. It meant that the product of NiS formed in the corrosion scale played a significant role in improving the stability and protectiveness of the corrosion scale. The increase of the NiS content promoted the formation of a staggered connected reticulate structure of the corrosion scale. This feature of the film structure was solid and continuous, which had a strong adhesion and was not readily peeled off from the coating surface or broken by mechanical action; meanwhile, it could hinder the corrosive medium to penetrate the corrosion scale [36], and thus slow down the mass transfer of corrosion species between the coating and the corrosion medium. Therefore, the corrosion rate of the coating decreased with the increase of the temperature.

**Figure 7:**
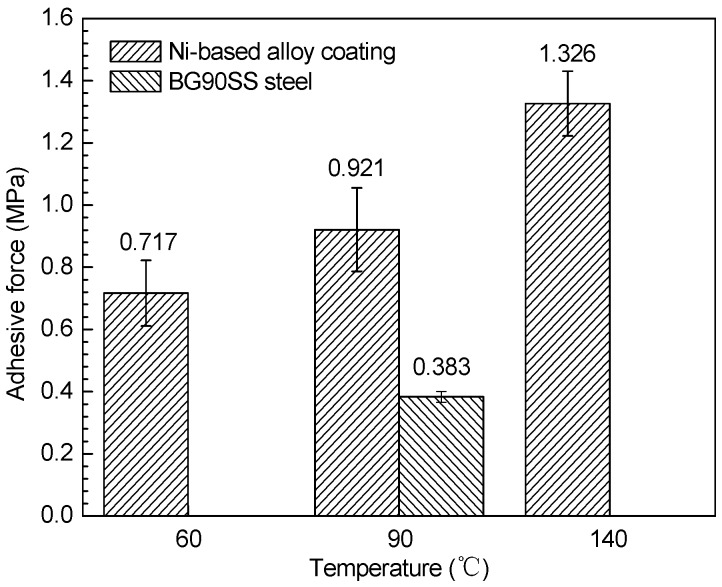
Adhesive force of the corrosion scale on the Ni-based alloy coating and BG90SS steel exposed to the H_2_S/CO_2_ environment for 168 h at different temperatures.

Apart from the chemical stability, the adhesive strength between the inside of the corrosion scale and the substrate under flowing conditions is also an important factor that influences the protective performance. Figure 7 shows the adhesive force of the corrosion scale on the Ni-based alloy coating and BG90SS steel at different temperatures, measured by means of the tensile method. As exhibited in the figure, the adhesive force of the corrosion scale on the Ni-based alloy coating increased with an increase in the temperature, and this was consistent with the results in Figure 5, where the corrosion rate decreased with the rise of temperature. The increase of the adhesive force of the corrosion scale was probably related to the formation of the reticulate structural scale. In addition, the interdiffusion of the corrosion products and the coating at the coating/scale interface might increase the adhesive force of the corrosion scale with the coating at a higher temperature [37]. In the H_2_S/CO_2_ environment with high temperature and pressure, the corrosion scale formed on the Ni-based alloy coating not only had a strong adhesive force but also had high acid resistance, providing good protection for the substrate. However, the adhesive force (0.383 MPa) of the corrosion scale on BG90SS steel was much lower than that (0.921 MPa) on the Ni-based alloy coating in the same environment, which justified the high corrosion rate of the former.

### 3.5. Effect of Immersion Time on the Characteristics of the Corrosion Scale

**Figure 8:**
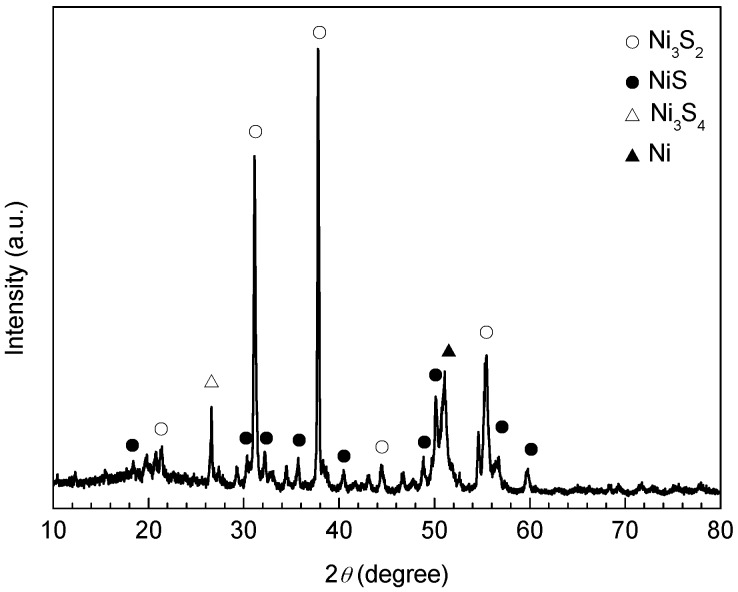
XRD spectra of the corrosion scale on the Ni-based alloy coating exposed to the H_2_S/CO_2_ environment for 360 h at 140 °C.

The corrosion rate of the Ni-based alloy coating after being corroded for 360 h at 140 °C was negative. This indicated that the stability and adhesive force of the corrosion scale were further improved. XRD analysis of the corrosion scale indicated that it was mainly composed of Ni_3_S_2_ and NiS, as well as small amounts of Ni_3_S_4_ and Ni (Figure 8).

**Figure 9:**
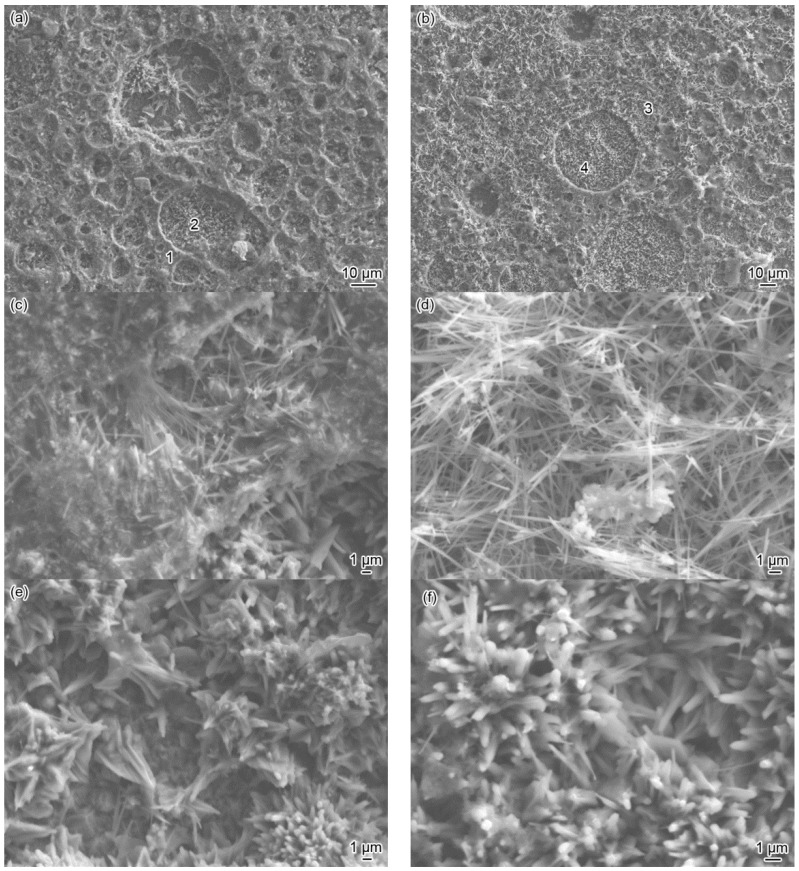
SEM surface morphologies of corrosion scales on the Ni-based alloy coating before (**a**,**c**,**e**) and after pickling (**b**,**d**,**f**) exposed to the H_2_S/CO_2_ environment for 360 h at 140 °C: (**c**,**e**) high magnification images of (**a**) denoted by 1 and 2, respectively and (**d**,**f**) high magnification images of (**b**) denoted by 3 and 4, respectively.

Figure 9 shows the SEM surface morphologies before and after removing the corrosion scales after being corroded for 360 h at 140 °C. It is obvious that the corrosion scale had a reticulate structure, with dense crater-shaped meshes of varying sizes (Figure 9a). The claylike corrosion products were stuffed between the needle-like corrosion products and were stacked at the rims of the craters to form “reticles” (Figure 9c). The needle-shaped or flaky corrosion products stacked in the craters to form many clusters (Figure 9e). After pickling, the reticulate structure of the corrosion products on the coating was still clear (Figure 9b). The locally magnified image shows that, the claylike corrosion products disappeared after pickling with only needle-shaped corrosion products stacked on the “reticles” (Figure 9d). EDS analysis revealed that the element ratio between Ni (49.72%) and S (50.28%) was about 1 in the needle-shaped corrosion products (Figure 9d), indicating that these needle-shaped corrosion products were NiS (Millerite) by combination with the results of the XRD analysis in Figure 8. However, the morphology of the cluster-shaped corrosion products had no obvious difference (Figure 9e,f), and their element ratio between Ni (59.67%) and S (40.33%) was about 1.5, indicating that the corrosion products were mainly Ni_3_S_2_ according to the results of the XRD analysis in Figure 8. Both needle-shaped NiS and cluster-shaped Ni_3_S_2_ had excellent chemical stability and functioned as the framework and main body of corrosion products, which were closely combined with the coating substrate, and collectively constituted the protective layer for the tubing steel substrate.

**Figure 10:**
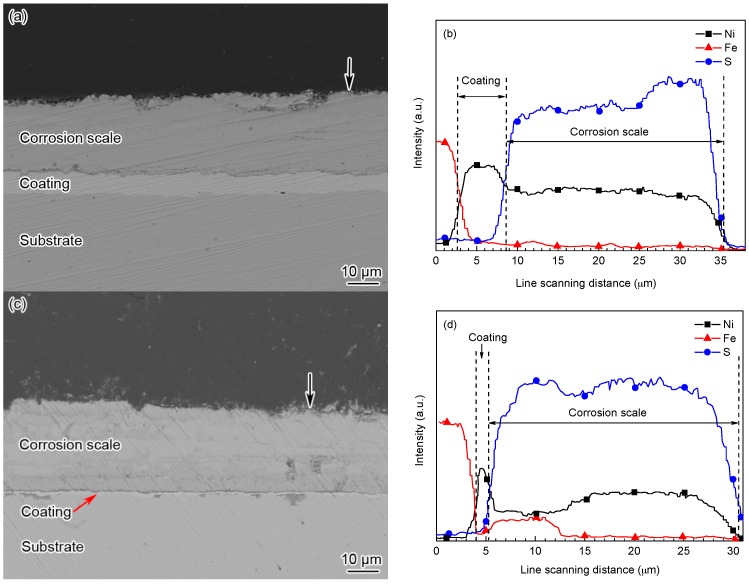
(**a**,**c**) SEM cross-sectional backscattered electron images and (**b**,**d**) elemental distributions in cross-sections of the Ni-based alloy coating and corrosion scale exposed to the H_2_S/CO_2_ environment at 140 °C: (**a**) after being corroded for 168 h; (**b**) denoted by the black arrow in (**a**); (**c**) after being corroded for 360 h and (**d**) denoted by the black arrow in (**c**).

Figure 10 shows the cross-sectional morphologies and elemental distributions of the Ni-based alloy coating after being corroded for 168 h and 360 h at 140 °C, respectively. Compared with the cross-sectional morphology of the original coating (Figure 1b), after being corroded, the outermost layer of the corrosion scale peeled off locally and the corrosion scale was apparently rougher (Figure 10a,c) than the original coating surface. However, the corrosion scale was homogeneous and compact, which was closely combined with the coating. After being corroded for 168 h, the remaining thickness of the coating was about 7 μm (Figure 10a), and most of the coating was oxidized into corrosion products. However, after being corroded for 360 h, the coating was almost fully converted into corrosion products, and the remaining thickness of the local coating was less than 1 μm (Figure 10c). It can be seen that the coating thickness was decreased by about 30 μm during the corrosion of the first 168 h, while it only decreased by about 4–5 μm after the subsequent corrosion of 192 h. The above results indicate that the corrosion rates calculated by using the weight loss method (Figure 5) cannot reveal the actual oxidation rate of the coating. The reason for the low weight loss was that the reticulate structural corrosion scale on the Ni-based alloy coating was stable, compact, and had strong adhesion, which could restrain the corrosion by retarding the reactant diffusion and consequently slow down the corrosion rate of steel, thus showing a good protectiveness.

The EDS line scanning analysis of the cross-sections of the corrosion scales revealed that the corrosion scale mainly contained Ni and S elements after being corroded for 168 h (Figure 10b). However, besides the Ni and S elements, a small amount of Fe element was also detected in the corrosion scale close to the substrate (Figure 10d). It can be seen that although the corrosion scale of the Ni-based alloy coating had good protectiveness, it cannot fully prevent the corrosive reactants from reaching the steel substrate, and consequently cause the corrosion of the tubing steel substrate.

## 4. Conclusions

The Ni-based alloy coating mainly consisted of Ni crystals and small amounts of NiW_2_P_3_ and Ni_2_P crystals, and the grain sizes of the Ni crystals were in the range of 12.1–20.3 nm. The nanocrystalline Ni-based alloy coating had no passivation in the H_2_S/CO_2_ environment. Both cathodic and anodic processes were simultaneously suppressed, and the corrosion current density and the corrosion rate calculated by the weight loss method were much lower than that of the BG90SS steel.

The Ni-based alloy coating presented a general corrosion morphology under the test temperatures. The corrosion rate calculated by the weight loss method decreased with an increase in the temperature, while the adhesive force of the corrosion scale on the Ni-based alloy coating increased. The corrosion rates calculated by the weight loss method cannot reveal the actual oxidation rate of the coating. The reason for the low weight loss was that the corrosion scale on the Ni-based alloy coating was stable, compact, and had strong adhesion.

The corrosion of the Ni-based alloy coating was mainly controlled by H_2_S corrosion, and the corrosion products, mainly consisting of Ni and S compounds, had excellent stability in acid solution. At 60 °C, the gel-like corrosion products with some pores were mainly composed of Ni_3_S_2_. At 90 °C, the corrosion scale stacked by tiny crystals with many small cauliflower-like bulges on the surface was mainly composed of Ni_3_S_2_ and a small amount of NiS. At 140 °C, the reticulate corrosion scale formed on the coating surface, which mainly consisted of Ni_3_S_2_, NiS, and a small amount of Ni_3_S_4_. With the increase of the temperature, the deposited morphology and composition of the corrosion products changed, the content of NiS in the corrosion scale increased, and the adhesive strength and stability of the corrosion scale improved. As the corrosion time prolonged, the Ni-based coating was thinned while the corrosion scale thickened. The corrosion scale closely combined with the coating, which can inhibit the mass transfer, but cannot fully prevent the corrosive reactants from reaching the substrate.

## Figures and Tables

**Table 1:** Composition of the plating solution of the Ni-based deposition [31].

Reagent	Concentration (g/L)	Operational Parameters
Nickel sulphate (NiSO_4_·6H_2_O)	130–230	Current density: 30–100 mA/cm^2^ Deposition time: 75 min Temperature: 50–80 °C pH: 3.4–5.4
Nickel Carbonate (NiCO_3_)	35–55	
Ammonium sulfate ((NH_4_)_2_SO_4_)	5–15	
Hydroxyethylidene (C_2_H_8_O_7_P_2_)	3–11	
Sodium Tungstate Dihydrate (Na_2_WO_4_·2H_2_O)	10–30	
Sodium allylsulfonate (C_3_H_5_SO_3_Na)	10–55	
1,4-butynediol (C_4_H_6_O_2_)	5–15	
Sodium citrate (Na_3_C_6_H_5_O_7_·2H_2_O)	32–66	
Sulfuric acid (H_2_SO_4_)	2–6	
Citric Acid (C_6_H_8_O_7_)	6–11	
Sodium dodecyl sulfate (C_12_H_25_SO_4_Na)	24–38	
Sodium hypophosphite (NaH_2_PO_2_·H_2_O)	8–26	

**Table 2:** Chemical composition of the formation water extracted from the gas condensate field.

Composition	NaCl	KCl	CaCl_2_	BaCl_2_·2H_2_O	MgCl_2_·6H_2_O	SrCl_2_·6H_2_O	NaBr
Concentration (g/L)	4.41	0.47	17.2	0.28	33.4	0.75	0.11

**Table 3:** Conditions of the weight loss tests.

Condition	Material	Temperature (°C)	Test Time (h)	CO_2_ (MPa)	H_2_S (MPa)	Flow Velocity (m/s)
1	Ni-based alloy coating	60	168	1.75	0.55	1.5
2	Ni-based alloy coating, BG90SS	90	168			
3	Ni-based alloy coating	140	168			
4	Ni-based alloy coating	140	360			

**Table 4:** Electrochemical parameters obtained from the polarization curves of the test materials in the simulated formation water at 25 °C and 0.1 MPa H_2_S/CO_2_.

Material	*E_corr_* (mV vs. SCE)	*i_corr_* (μA/cm^2^)	*b_a_* (mV/decade)	*b_c_* (mV/decade)
Ni-based alloy coating	−525	2.48	97.6	−389
BG90SS steel	−704	14.3	64.9	−251
